# Transcriptomic profiling of cartilage ageing

**DOI:** 10.1016/j.gdata.2014.03.001

**Published:** 2014-03-19

**Authors:** Mandy Jayne Peffers, Xuan Liu, Peter David Clegg

**Affiliations:** a Comparative Musculoskeletal Biology, Institute of Ageing and Chronic Disease, University of Liverpool, Leahurst, Chester High Road, Neston, Wirral CH64 7TE, United Kingdom; b Centre for Genomic Research, Institute of Integrative Biology, Biosciences Building, Crown Street, University of Liverpool, Liverpool L69 7ZB, United Kingdom

**Keywords:** Cartilage, Ageing, RNASeq, Small non-coding RNA, Extracellular matrix

## Abstract

The musculoskeletal system is severely affected by the ageing process, with many tissues undergoing changes that lead to loss of function and frailty. Articular cartilage is susceptible to age related diseases, such as osteoarthritis. Applying RNA-Seq to young and old equine cartilage, we identified an over-representation of genes with reduced expression relating to extracellular matrix, degradative proteases, matrix synthetic enzymes, cytokines and growth factors in cartilage from older donors. Here we describe the contents and quality controls in detail for the gene expression and related results published by Peffers and colleagues in Arthritis Research and Therapy 2013 associated with the data uploaded to ArrayExpress (E-MTAB-1386).

**Table:** 

Specifications	
Organism/cell line/tissue	*Equus caballus*
Sex	Castrated males
Sequencer or array type	Illumina HiSeq 2000
Data format	Raw: FASTQ txt files
Experimental factors	Age: young (4 years old) and old (> 15 years old)
Experimental features	RNA-Seq dataset of young and old equine cartilage
Consent	Samples were collected as a by-product of the agricultural industry. Specifically the Animal (Scientific Procedures) Act 1986, Schedule 2, does not define collection from these sources as scientific procedures. Ethical approval was therefore not required for this study.
Sample source location	Liverpool, UK

## Direct link to deposited data

Deposited data can be found at: http://www.ebi.ac.uk/arrayexpress/experiments/E-MTAB-1386/.

## Experimental design, materials and methods

### Sample collection and preparation

Full thickness equine cartilage from the entire surface of macroscopically normal metacarpophalangeal joints of eight horses (four young horses, aged four years old and four old horses, greater than 15 years old) was collected from an abattoir. Horses selected were non-thoroughbred leisure horses in this study [1]. Macroscopic scoring of the metacarpophalangeal joint was measured using a macroscopic grading system as previously described [2] and samples with no macroscopic perturbations were selected.

### Gene expression analysis

Cartilage from both articular condyles was removed under sterile conditions into RNAlater (Sigma-Aldrich, Dorset, UK) according to manufacturer's instructions. Cartilage was pulverised into a powder with a dismembranator (Mikro-S, Sartorius, Melsungen, Germany) following freezing in liquid nitrogen prior to addition of Tri Reagent (Ambion, Warrington, UK). RNA was extracted using the guanidium–thiocyanate–phenol–chloroform technique, as described previously [3]. RNA was purified using RNeasy spin columns (Qiagen, Crawley, UK) with on-column DNase treatment (Ambion, Warrington, UK). RNA was quantified using a Nanodrop ND-100 spectrophotometer (Labtech, East Sussex, UK) and assessed for purity by UV absorbance measurements at 260 nm and 280 nm. Eight libraries representing four animals from two groups, young and old (n = 4 young and n = 4 old), were prepared. Total RNA was analysed by the Centre for Genomic Research, University of Liverpool, for RNA-Seq library preparation and sequencing using the Illumina HiSeq 2000 platform. Total RNA integrity was confirmed using an Agilent 2100 Bioanalyzer (Agilent Technologies, Santa Clara, CA, USA). Ribosomal RNA (rRNA) was depleted from 8 total RNA samples using the Ribo-Zero™ rRNA Removal Kit (Human/Mouse/Rat EpiCentre, Madison, USA) following the manufacturer's instructions. cDNA libraries were prepared with the ScriptSeq v2 RNA-Seq library preparation kit (Epicentre, Madison, USA) using 50 ng rRNA depleted RNA as starting material and following the manufacturer's protocols. Briefly, rRNA depleted sample was fragmented using an RNA fragmentation solution prior to cDNA synthesis. Fragment size of the final libraries and pooled libraries was confirmed using the Agilent 2100 Bioanalyzer software in smear analysis function. Fragmented RNA was reverse transcribed using random-sequence primers containing a tagging sequence at their 5′ ends. 3′ tagging was accomplished using the Terminal-Tagging Oligo (TTO) which features a random nucleotide sequence at its 3′ end, a tagging sequence at its 5′ end and a 3′-blocking group on the 3′terminal nucleotide. The TTO was randomly annealed to the cDNA, including to the 3′ end of the cDNA. Purification of the di-tagged cDNA was undertaken with AMPure™ XP (Agencourt, Beckmann-Coulter, USA). The di-tagged cDNA underwent 15 cycles of amplification using PCR primer pairs that were annealed to the tagging sequences of the ditagged cDNA. Excess nucleotides and PCR primers were removed from the library using AMPure™ XP (Agencourt, Beckman-Coulter, USA). The final pooled library was diluted to 8 pmol before hybridisation. The dilute library (120 μl) was hybridised on each of 3 HiSeq lanes.

### Data processing and normalisation

The 100 bp paired-end raw reads in FASTQ format obtained by RNA-Seq were compiled using manufacturer provided pipeline software CASAVA 1.8.2. Quality control of the reads in each lane was undertaken in FASTQC [4]. Between 116 and 235 million reads were obtained per sample. Low quality reads were eliminated resulting in 7–58 million mapped reads (equal to 6.5–35% of the total reads). In total, 3–49 million uniquely mapped read pairs were obtained per sample after aligning to the reference sequence of the equine genome (Equus caballus; EquCab2.56.pep, downloaded from ftp://ftp.ensembl.org/pub/current_fasta/equus_caballus/pep/) (Table 1) with TOPHAT 1.3.2 using default settings, except for the option “-g 1”. The option “-g 1” instructs TopHat to allow a maximum of 1 alignment to the reference for a given read, choosing the alignment with the best alignment scores if there is more than 1 or discarding the read if there is more than 1 equally good alignment. Read counts per gene were calculated using HTSeq-count (http://www-huber.embl.de/users/anders/HTSeq/doc/count.html) with default settings. Differential gene expression analysis was applied to the read count data for reads mapped to annotated genes using the R (version 2.15.1) Bioconductor package edgeR (version 2.13.0) [5]. EdgeR models data as a negative binomial distribution to account for biological and technical variation using a generalisation of the Poisson distribution model. Prior to assessing differential expression, data were normalised across libraries using the trimmed mean of M-values normalisation method [6]. Genes were deemed differentially expressed with a Benjamini–Hochberg false discovery rate (FDR)-corrected p-value < 0.05 and fold change ≥ 1.4log_2_ [7] using a generalised linear model (GLM) likelihood ratio test. This represents a 50% linear fold change i.e. log_2_ 1.4 = 0.5 or 50%. All sequence data produced in this study has been submitted to NCBI GEO under Array Express accession number E-MTAB-1386.

The number of genes per read was normalised to ‘reads per kilo base of exon model per million mappable reads’ (RPKM); therefore the values were considered the final expression level for each gene [8]. In total 16,635 genes were expressed in cartilage, which represented 66% of the equine genome.

The expression of 396 transcribed elements including mRNAs, small non-coding RNAs, pseudogenes, and a single microRNA was significantly different in old compared to young cartilage. Of these, 93 were at higher levels in the older cartilage and 303 were at lower levels in the older cartilage.

## Discussion

Here we describe a unique dataset of equine ageing cartilage. This dataset is comprised of whole transcriptome gene expression profiling data derived using the Illumina HiSeq 2000. We demonstrated differential expression with ageing of protein-coding and non-protein coding RNAs including small non-coding RNAs, pseudogenes and microRNA. The dataset was used in studies published recently and is the first published study to use next-generation sequencing data to interrogate ageing cartilage. Results from the data have increased our understanding of cartilage ageing.

## Figures and Tables

**Table 1:** Uniquely mapped reads aligned to the equine genome.

Reads category	Old 1	Old 2	Old 3	Old 4	Young 1	Young 2	Young 3	Young 4
Total reads	1.E + 08	1.E + 08	2.E + 08	1.E + 08	2.E + 08	2.E + 08	1.E + 08	1.E + 08
Mapped reads	1.E + 07	1.E + 07	2.E + 07	1.E + 07	6.E + 07	2.E + 07	8.E + 06	5.E + 07
Failed mapped reads	1.E + 08	1.E + 08	2.E + 08	1.E + 08	1.E + 08	2.E + 08	1.E + 08	1.E + 08
Properly paired mapped	6.E + 06	6.E + 06	8.E + 06	6.E + 06	5.E + 07	8.E + 06	4.E + 06	4.E + 07
